# Nature Appropriation and Associations with Population Health in Canada’s Largest Cities

**DOI:** 10.3390/ijerph10041268

**Published:** 2013-03-26

**Authors:** Daniel Rainham, Rory Cantwell, Timothy Jason

**Affiliations:** 1 Environmental Science, Faculty of Science, Dalhousie University, Halifax, Nova Scotia B3H 4R2, Canada; E-Mail: tjd@dal.ca; 2 Atlantic Health Promotion Research Centre, Dalhousie University, Halifax, Nova Scotia B3J 3T1, Canada; 3 Department of Natural Resource Sciences, McGill University, Montreal, Quebec H9X 3V9, Canada; E-Mail: rory.cantwell@mail.mcgill.ca

**Keywords:** sustainability, population health, natural capital, ecological footprint analysis, nature appropriation

## Abstract

Earth is a finite system with a limited supply of resources. As the human population grows, so does the appropriation of Earth’s natural capital, thereby exacerbating environmental concerns such as biodiversity loss, increased pollution, deforestation and global warming. Such concerns will negatively impact human health although it is widely believed that improving socio-economic circumstances will help to ameliorate environmental impacts and improve health outcomes. However, this belief does not explicitly acknowledge the fact that improvements in socio-economic position are reliant on increased inputs from nature. Gains in population health, particularly through economic means, are disconnected from the appropriation of nature to create wealth so that health gains become unsustainable. The current study investigated the sustainability of human population health in Canada with regard to resource consumption or “ecological footprints” (*i.e.*, the resources required to sustain a given population). Ecological footprints of the 20 largest Canadian cities, along with several important determinants of health such as income and education, were statistically compared with corresponding indicators of human population health outcomes. A significant positive relationship was found between ecological footprints and life expectancy, as well as a significant negative relationship between ecological footprints and the prevalence of high blood pressure. Results suggest that increased appropriation of nature is linked to improved health outcomes. To prevent environmental degradation from excessive appropriation of natural resources will require the development of health promotion strategies that are de-coupled from ever-increasing and unsustainable resource use. Efforts to promote population health should focus on health benefits achieved from a lifestyle based on significantly reduced consumption of natural resources.

## 1. Introduction

Sustainability is of fundamental importance to humanity especially as anthropogenic activities continue to debilitate the environmental conditions required to support and sustain life. Here we define sustainability as a form of equitable human development that does not jeopardize the opportunity for all life to flourish, now and in the future. The appropriation of Earth’s natural capital by a rapidly growing global population is occurring in an unsustainable fashion. Estimates show that humans appropriate large proportions of present net primary production in the biosphere [1]. Global fisheries have deteriorated significantly due to over-fishing and habitat destruction [2,3], and rising energy demands have exacerbated conflict over energy sources, as well as the release of CO_2_ and other anthropogenic pollutants associated with global warming [4].

Several indicators have been proposed to estimate human impact on natural resources, including: the living planet index [5], percent threatened species, current forest as a percentage of original forest, and percentage of land disturbed by human activity [6,7]. Another, the ecological footprint, estimates the amount of biologically productive land, expressed in global hectares *per capita* (Gha/capita), required to sustain human demands over the course of a year [8,9]. Ecological footprint analysis (EFA) results in a value of the comparison between resource demands and available biocapacity derived from six components: (1) energy land or the area of forest theoretically required to absorb CO_2_ emissions, (2) area of crop land, (3) area of grazing land, (4) forest land or the area of forest consumed for wood and paper products, (5) sea space or the area of ocean required to supply marine fish and seafood resources, and (6) built area or the area of land allocated for housing and infrastructure. Recent EFA research suggests that the available global biocapacity supply is approximately 11.2 billion hectares (ha) or 1.7 ha *per* person [10]. This value assumes an equal distribution of natural resources and land for human use only. The reality, however, is that the global supply of Earth’s natural capital is disproportionately consumed by more industrial, wealthier nations [11,12]. Indeed, current rates of consumption will eventually exhaust natural resources leading to an ecological deficit for future generations.

Economic development and wealth have been generally achieved through the usurpation of natural capital. For example, mining, fishing, forestry, agricultural, and energy production activities all contribute substantially to a nation’s GDP while depleting renewable and non-renewable resources [13]. Prior research has documented an association between national wealth and consumption rates [14,15,16], as well as population health [17,18], although the latter association remains debatable [19]. In other words, there is a good association between natural capital consumption and benefits to population health. More wealthy nations use Earth’s resources to generate wealth required to afford better and more universal health care, procure more energy, secure safe and reliable food supplies, and provide more sanitary conditions [20], all of which enhance population health. Understanding the relationship between levels of nature appropriation and health outcomes is important if population health is to be maintained or improved indefinitely. Research on the country-level relationship between consumption and population health found an inverse relationship between environmental sustainability indicators and human health outcomes, suggesting that achieving health, at least in more industrialized states, requires unsustainable levels of nature appropriation [6,21,22]. However, the analyses typically are global in scale and there is uncertainty as to whether the relationship between consumption and health holds when assessed at other scales. Moreover, the relationship between natural capital appropriation and human health remains a relatively unexplored topic.

Canada presents an unusual global case in that its available biocapacity is two times greater than current population consumption patterns—an ecological surplus. Nature consumption rates appear sustainable yet realistically are neither reasonable nor sustainable when situated in a global context. The purpose of the current study is to ascertain the relationship between nature appropriation and human health within a country where there is much less variation in overall patterns of nature appropriation. Does the relationship between nature appropriation and population health hold true within a country with strong social and income support mechanisms, and where consumption patterns exhibit little variation (compared to global differences) from place to place? To properly address this research question, several indicators of human health were assessed in relation to natural capital consumption rates at the municipal (local) level. Specifically, ecological footprints of the twenty most highly populated cities in Canada were evaluated against six human health indicators in order to determine the impact of consumption on human population health.

## 2. Methods

This ecological study statistically evaluates the association between natural capital appropriation and several indicators of population health. Nature appropriation was calculated using ecological footprint analysis. The ecological footprint (EF) is a value of land area equivalents required to support the consumption patterns of defined population. Table 1 reveals footprint estimates for the 20 largest Canadian cities [23]. Several health indicators were included in the analysis since there is little confirmatory evidence regarding health outcomes most affected by natural capital appropriation. Three additional determinants of health (income, education and smoking status) were included in the analysis as covariates also known to strongly influence variations in health status. Table 2 provides a summary of the variables used in the analysis and their sources.

Univariate analysis revealed some non-normality in the data. Scatterplots of the relationship between ecological footprint and several health outcomes exhibited non-linearity. Variables were transformed where required for inclusion into multivariate regression models. Best subsets regression analyses were performed to assess the influence of ecological footprint in explaining variations in health controlling for socioeconomic status and smoking at the municipal level. The best subsets approach compares all possible regression models using a specified set of predictors, and provides the best-fitting models that contain one or more covariates. Inclusion of covariates in the final model was according to the lowest possible variance inflation factors (VIF). If the addition of a covariate resulted in the amplification of VIF, then it was removed from the analysis with the intention of reducing the potential for multicollinearity and ensuring stability of model estimates. Minitab^®^ Release 14 Statistical Software was employed for all analyses.

**Table 1:** Ecological footprints (EF) for the twenty most highly populated Canadian cities.

City	EF (ha)	City	EF (ha)
Vancouver	7.7	Niagara	6.8
Calgary	9.8	Hamilton	7.3
Edmonton	9.4	Halton	8.9
Regina	7.4	Peel	7.8
Saskatoon	7.2	York	8.2
Winnipeg	7.1	Toronto	7.3
Windsor	7.3	Kingston	7.1
London	6.9	Ottawa	8.5
Waterloo	7.4	Quebec	6.8
Greater Sudbury	6.8	Halifax	7.8

ha: hectares *per capita*.

**Table 2:** Summary and sources of analytical variables.

Indicator	Source	Year	Calculation/Measure
**Nature Appropriation**			
Ecological Footprint *	Federation of Canadian Municipalities	2005	Hectare/capita
**Population Health**			
Life Expectancy	Statistics Canada, Vital Statistics, Death Database, and Demography Division	2005	3 year average between 2000–2002; years.
Infant Mortality	Statistics Canada, Vital Statistics, Birth and Death Databases	2005	3 year average in 2000–2002; Expressed as deaths/1,000 live births
Premature Death	Statistics Canada, Vital Statistics, Death Database, and Demography Division	2005	3 year average of years of life lost due to premature death (<75 years); rate per 100,000 people
High Blood Pressure	Statistics Canada, Canadian Community Health Survey, 2003, 2000/01, health file	2004	Population aged 12 and over who reported a diagnosis of high blood pressure
Circulatory Disease Mortality	Statistics Canada, Vital Statistics, Death Database, and Demography Division	2005	3 year average of years of life lost due to circulatory disease; rate per 100,000 people
Body Mass Index: Obese	Statistics Canada, Canadian Community Health Survey, 2003, 2000/01, health file	2004	Percentage of population that is obese (BMI > 30.0)
**Covariates**			
Income	Statistics Canada, 1996 and 2001 Census	2001	Average income for persons aged 15 and over
Education	Statistics Canada, 2001 Census of Population	2002	Percentage of the 20–64 year age group with a college certificate or diploma
Daily Smoker	Statistics Canada, Canadian Community Health Survey, 2000/2001	2001	Percentage of the population that reports to smoke daily

* The quality of data required for the calculation of ecological footprint values varies among municipalities. The Ville de Montreal chose not to participate in the process of calculating EF values. Values used in this study did not incorporate uncertainty in the analysis.

## 3. Results

Descriptive statistics for municipal ecological footprints (EF) and population health indicators are presented in Table 3.These data characterize urban populations with relatively high standards of living and human development. Canada is known internationally for government spending on social support programs and accessibility to high quality health care and public health programs. Table 4 provides a summary of the regression results presented for each population level health outcome.

**Table 3:** Descriptive statistics for study variables.

Variable	Mean	Median	Standard Deviation	Minimum	Maximum
EF (ha)	7.7	7.4	0.9	6.8	9.8
Income (CDN $)	20,960	20,419	2,308	18,202	27,462
Education (%)	24.2	22.6	5.9	14.9	37.3
Daily Smoking (%)	20.5	20.8	3.3	13.4	28.1
LE (years)	79.7	79.6	1.1	77.3	81.6
IM (/1000 live births)	5.6	5.7	0.9	3.8	8.0
PMD (years life lost)	4,913	5,008	828	3,279	6,723
HBP (%)	14.2	14.05	1.8	11.0	17.3
CDM (years life lost)	203	208	26.3	164	262
BMI (%)	14.6	14.7	3.0	6.1	18.4

EF: Ecological footprint in hectares; LE: life expectancy; IM: Infant mortality; PMD: Premature death; HBP: High blood pressure; CDM: Circulatory disease mortality; BMI: Body mass index.

**Table 4:** Regression model results of the association between EF and health outcomes.

Outcome	Significant Covariates	*F* (*p*-value)	*R^2^* (%)
Life Expectancy	+ Ecological Footprint − Smoking	22.0 (<0.001)	69
Premature Death	+ Smoking	11.0 (<0.001)	61
High Blood Pressure	− Ecological Footprint − Education	13.0 (<0.001)	55
Circulatory Disease Mortality	− Education	11.0 (<0.001)	50
Body Mass Index	+ Smoking	9.8 (<0.001)	65
Infant Mortality	-	4.8 (>0.05) *	37

* Non-significant model.

### 3.1. Life Expectancy (LE) and EF

**Figure 1:**
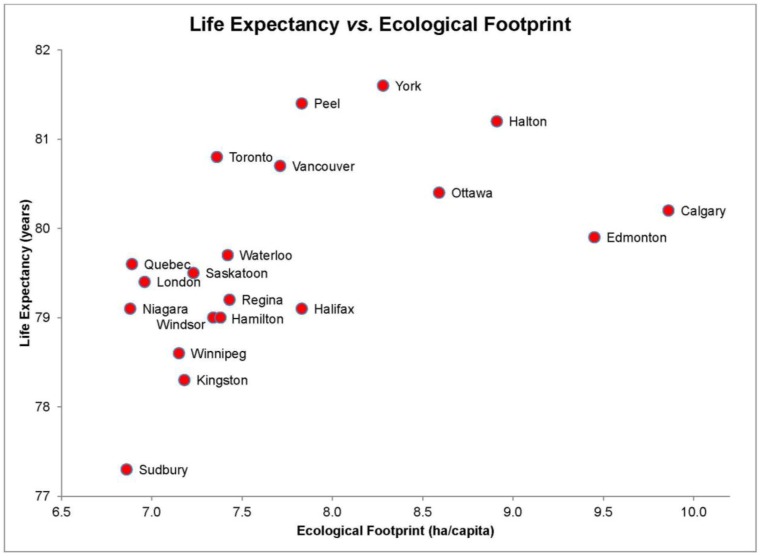
Life expectancy (years) and Ecological Footprints (ha/capita) of the 20 most highly populated cities in Canada.

The scatter plot for life expectancy and EF revealed a non-linear relationship (Figure 1). Specifically, life expectancy initially increases as a function of increasing EFs, plateaus, and then decreases, which suggests the presence of diminishing life expectancy associated with high levels of natural capital consumption relative to other Canadian cities. According to the scatter plot, life expectancy is highest in York with an EF of 8.28 ha/capita and lowest in Sudbury with an EF of 6.87 ha/capita. In bivariate tests, statistically significant positive correlations were found for EF (*r_s_* = 0.66, *p* < 0.05), income (*r_s_* = 0.52, *p* < 0.05) and education (*r_s_* = 0.73, *p* < 0.05) and life expectancy, and daily smoking rates (*r_s_* = −0.62, *p* < 0.05).

The final multiple regression model for life expectancy included two predictors (EF and daily smoking rate) yielded a significant relationship, *F*(2, 17) = 22, *p* < 0.001 and an *R^2^* of 0.69 indicating that approximately 69% of the variability in life expectancy is predicted by this model. EF individually accounted for a significant percentage of the variability in life expectancy (*t*(17) = 3.1, *p* < 0.05). According to these results, EF is associated with longer life expectancy.

### 3.2. Premature Death (PMD) and EF

**Figure 2:**
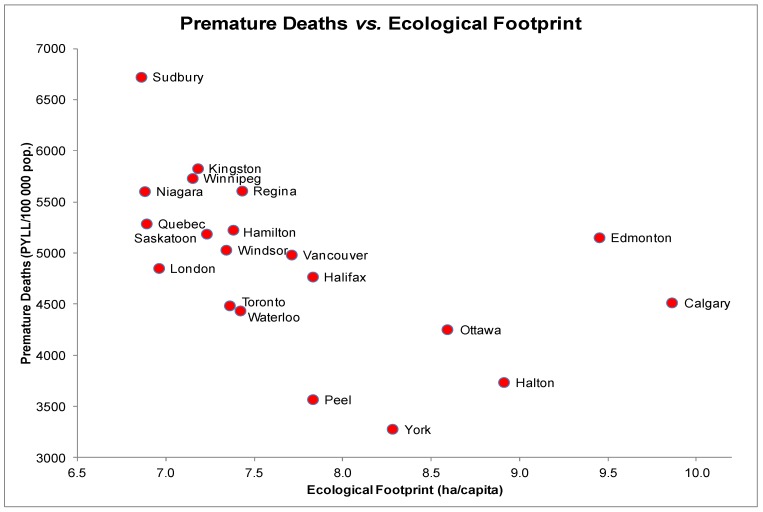
Premature Deaths (PYLL/100,000) and Ecological Footprints (ha/capita) of the 20 most highly populated cities in Canada.

The scatter plot for PMD and EF indicated a non-linear relationship with initially decreasing PMD rates followed by a gradual increase (Figure 2). PMD rates are highest in Sudbury with an EF of 6.87 ha/capita and lowest in York with an EF of 8.28 ha/capita. Correlation testing produced a significant negative association between PMD and EFs (*r_s_* = −0.68, *p* < 0.05). Scatter plots of other health measures with PMD revealed a linear distribution. Spearman correlation tests yielded significant negative correlations between PMD and income (*r_s_* = −0.62, *p* < 0.05) as well as PMD and education (*r_s_* = −0.64, *p* < 0.05). A significant positive correlation was also observed between PMD and daily smoking rates (*r_s_* = 0.61, *p* < 0.05).

A multiple regression test on PMD including four predictors (EF, income, education and daily smoking rate) yielded a significant relationship, *F*(3, 16) = 11, *p* < 0.001 and an *R*^2^ of 0.61 indicating that approximately 61% of the variability in PMD is predicted by this model. EF individually accounted for a non-significant percentage of the variability in PMD (*t*(16) = −1.2, *p* = 0.24) due to probable mediation of the other three predictors. The strongest predictor was daily smoking rate, which accounted for a significant percentage of the variability in PMD (*t*(16) = 3.0, *p* < 0.05).

Despite the significant zero-order correlation between PMD and EF, the relationship is likely governed by the robust relationships between income, education and EF. The analysis shows that higher incomes and education are strongly associated with lower rates of PMD, and that they are also associated with a larger EF.

### 3.3. High Blood Pressure (HBP) and EF

**Figure 3:**
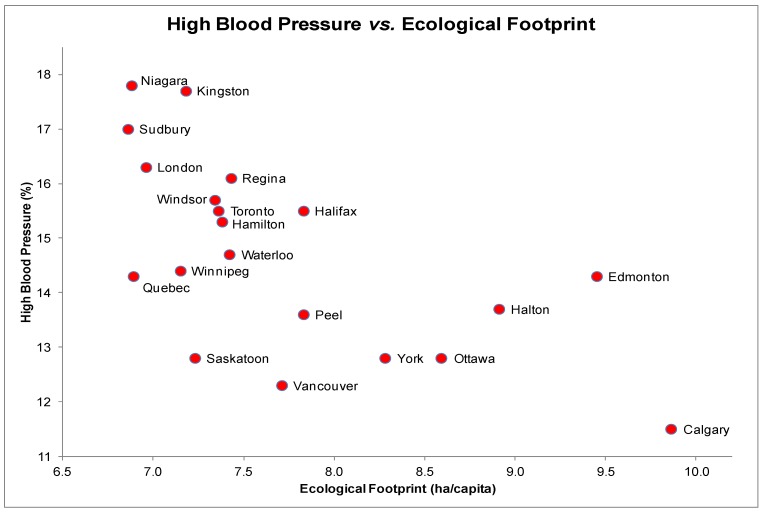
High Blood Pressure (%) and Ecological Footprints (ha/capita) of the 20 most highly populated cities in Canada.

The scatter plot for HBP and EF showed a non-linear relationship (Figure 3) with most data points clustering in the high blood pressure-low EF area of the graph. Niagara (EF = 6.88 ha/capita) was associated with the most self-reported HBP diagnoses while Calgary (EF = 9.86 ha/capita) was associated with the fewest self-reported HBP diagnoses. A significant negative association was found between HBP and EFs (*r_s_* = −0.66, *p* < 0.05). Each scatter plot of the other health determinants and HBP revealed a linear distribution. Additional correlation tests yielded significant negative correlations between HBP and income (*r_s_* = −0.52, *p* < 0.05) and HBP and education (*r_s_* = −0.69, *p* < 0.05).

A regression model with HBP as the dependent variable included terms for EF, income and education and yielded a statistically significant relationship, *F*(2, 17) = 13, *p* < 0.001, with an *R*^2^ of 0.55 indicating that approximately 55% of the variability in self-reported HBP diagnoses is predicted by the full model. Of the four covariates, the negative coefficients for EF (*t*(17) = −2.5, *p* < 0.05) and education were statistically significant. Higher EF values are significantly and inversely associated with rates of self-reported HBP diagnoses even after adjustment for socioeconomic factors such as income and education.

### 3.4. Circulatory Disease Mortality (CM) and EF

**Figure 4:**
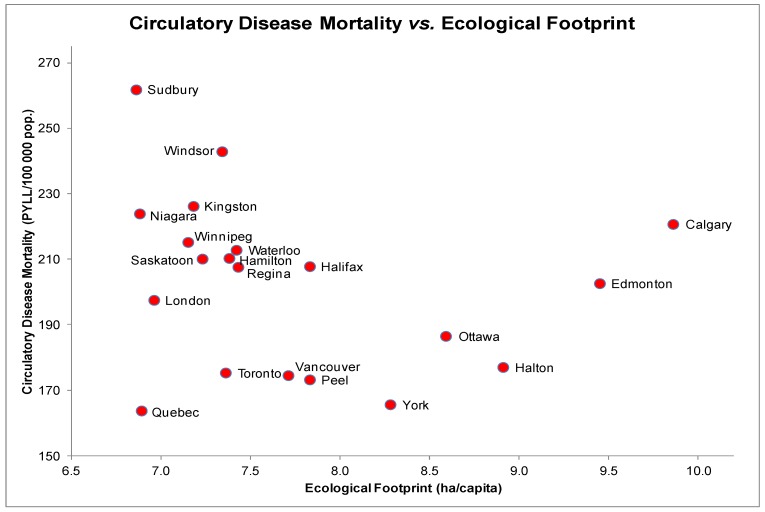
Circulatory Disease Mortality (PYLL/100,000) and Ecological Footprints (ha/capita) of the 20 most highly populated cities in Canada.

The scatter plot for CM and EF indicated a non-linear relationship (Figure 4). Interestingly, despite similar EFs, Sudbury is associated with higher rates of PMD due to circulatory disease than both Niagara and Quebec. There was no evidence of a significant association between CM and EF (*r_s_* = −0.37, *p* = 0.11). Each scatter plot of other determinants and CM revealed a linear distribution. Additional tests yielded significant correlations between CM and education (*r_s_* = −0.71, *p* < 0.05) and CM and daily smoking rates (*r_s_* = 0.47, *p* < 0.05).

The regression model with CM as the dependent variable included EF, education and daily smoking rates as covariates. These covariates explained a significant proportion of the variance in CM, *F*(2, 17) = 11.0, *p* < 0.001, with an *R^2^* = 0.50 indicating that approximately half of the variability in CM is predicted by this model. EF accounted for a non-significant percentage of the variability in CM (*t*(17) = 0.66, *p* = 0.52), likely due to collinearity in the model and a strong correlation between EF and education.

### 3.5. Body Mass Index (BMI) and EF

**Figure 5:**
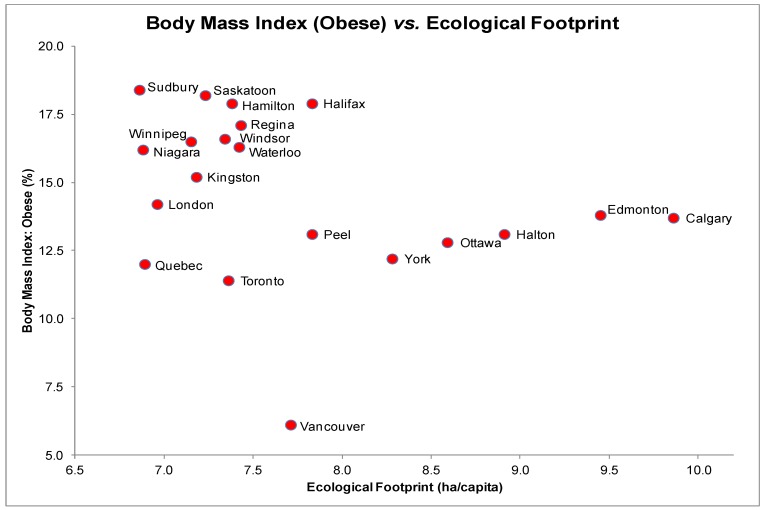
Obesity (% BMI > 30) and Ecological Footprints (ha/capita) of the 20 most highly populated cities in Canada.

The scatter plot for BMI and EF presented a linear yet statistically non-significant relationship (Figure 5), *r_s_* = −0.36, *p* = 0.12. Despite having a moderate level of ecological consumption (EF of 7.71 ha/capita), Vancouver also had a relatively low prevalence of obesity. Each scatter plot of other health determinants and BMI revealed a linear distribution. Correlation tests yielded significant relationships between BMI and education (*r_s_* = −0.73, *p* < 0.05) as well as BMI and daily smoking rates (*r_s_* = 0.68, *p* < 0.05).

A multiple regression test on BMI including four predictors (EF, income, education and daily smoking rates) yielded a significant relationship, *F*(4, 15) = 9.8, *p* < 0.001 and an *R^2^* of 0.65 indicating that approximately 65% of the variation in obesity rates can be explained by this model. EF individually accounted for a non-significant percentage of the variability in BMI (*t*(15) = −0.91, *p* = 0.38) suggesting the presence of multicollinearity. The only significant predictor was daily smoking rate, which accounted for a significant percentage of the variability in BMI (*t*(15) = 2.2, *p* < 0.05).

### 3.6. Infant Mortality (IM) and EF

**Figure 6:**
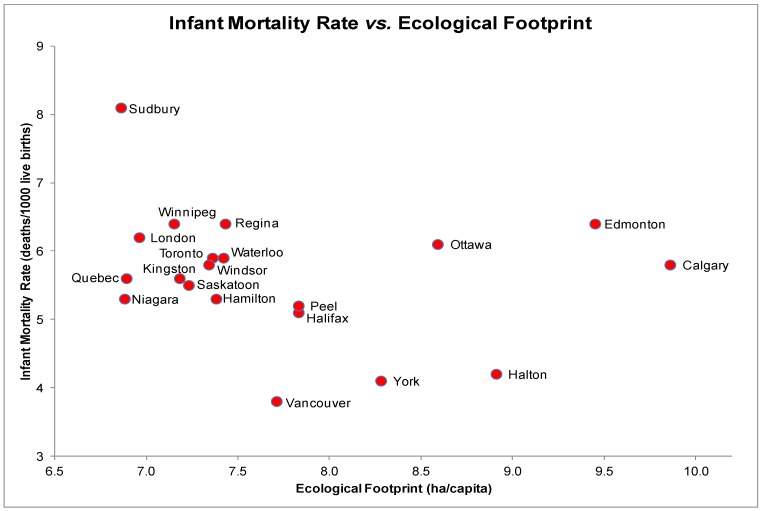
Infant Mortality Rate (deaths/1,000 live births) and Ecological Footprints (ha/capita) of the 20 most highly populated cities in Canada.

The scatter plot for IM and EF indicated a linear yet statistically non-significant relationship (Figure 6), *r_s_* = −0.29, *p* = 0.22. IM rates were highest in Sudbury with an EF of 6.87 ha/capita and lowest in Vancouver with an EF of 7.71 ha/capita. Each scatter plot of other health determinants and IM revealed a linear distribution and statistically non-significant relationship.

A multiple regression test on IM including three predictors (EF, education and daily smoking rates) yielded a significant relationship, *F*(3, 16) = 4.8, *p* > 0.05 and an *R*^2^ of 0.37 indicating that only about one-third of the variability in IM rates is predicted by this model. EF individually accounted for a non-significant percentage of the variability in IM (*t*(16) = −0.32, *p* = 0.75. However, no predictor yielded a significant coefficient in the model.

## 4. Discussion

This study relates ecological footprint data to six different human population health indicators in Canada’s twenty most highly populated cities. Results demonstrated a significant positive relationship between life expectancy and ecological footprint as well as a significant negative relationship between rates of high blood pressure and ecological footprint data after controlling for additional key health determinants. Even after controlling for level of education, income and smoking rates, the degree of nature appropriation explained a significant proportion of variation in life expectancy and rates of high blood pressure. At the municipal level, the amount of nature appropriation was less important in explaining variations in body mass index, circulatory disease, infant mortality and premature death; these measures of health were more likely to be associated with smoking rates, income and education.

This is the first study to show an ecological association between life expectancy rates of high blood pressure and the consumption of nature at the local level. The results of this study align with previous research on the relationship between levels of natural capital appropriation and health outcomes conducted at the national or country-level scale. For example, Rainham and McDowell reported a positive non-linear relationship between ecological footprint and disability-adjusted life expectancy even after adjustment for *per capita* GDP among 157 countries and a total population of 5.7 billion [21]. Other studies have also reported a relationship between nature appropriation and health outcomes (usually life expectancy or mortality measures) although the strength and significance of the relationships declined after accounting for income and other important social determinants of health [6,22]. The present analysis is differentiated from previous studies by analyzing the role of nature appropriation in explaining variations in up to six health measures, including life expectancy, and through adjustment by inclusion of several additional socioeconomic predictors of health in the regression models. Moreover, the range of variation in ecological footprint values within Canada is narrow when compared to global values. Our findings add support to the hypothesis that the level of nature appropriation is an important macro-level environmental determinant of population health. Due to the exploratory nature of the study, several health outcomes were included in the analysis as there is little prior confirmatory evidence regarding the influence of nature appropriation on health.

An interesting outcome of the current study is that increasing natural capital appropriation results in improved health outcomes to a certain point, at which there is indication that excess appropriation leads to a reversal of health gains. For example, Rainham and McDowell reported a positive non-linear relationship between natural capital appropriation and improved health outcomes in a large international sample [21]. Similar non-linear relationships were observed among Canadian cities suggesting that once enough resources are available to satisfy requirements for health further appropriation of natural resources has little effect on improvements to health. For health outcomes that were significantly influenced by the consumption of nature, the relationship revealed a pattern of diminishing returns; thus, increasing ecological footprints improve health and life expectancy to a point after which continued (or excessive consumption) appears to negatively affect health. It is interesting to note that Edmonton and Calgary, two cities with the highest EF values, lower life expectancies and higher rates of mortality, have the highest residential energy use *per capita*, second highest GHG emissions *per capita*, and have the highest number of vehicle km traveled *per capita*. The number of vehicle km traveled is directly associated with air pollutant concentrations and rates of overweight and obesity [24]. In addition, the effect on health of increasing natural capital appropriation is considerably greater for cities with lower rates of nature appropriation than for cities with higher rates, and echoes the concept of diminishing returns. A city with a low ecological footprint value may experience substantial health gains from increasing natural capital appropriation though industrial and commercial means. Conversely, cities with already high ecological footprints will experience little health benefit (or possibly reductions in health benefits) as a result of increased natural capital appropriation. The marginal benefit of increased consumption is much greater at lower EFs whereas the marginal cost of increased consumption is greater at higher EFs.

Although income is significantly associated with several health outcomes in bivariate (correlation) analyses, it did not explain a significant proportion of the variation among any of the health outcomes we evaluated, in the context of a multiple regression analysis, which also included ecological footprint as a predictor. Previous studies, as well as studies of the relationship between income and health more generally, have found that health improvements are associated with increasing income, although in a non-linear fashion, similar to the shape of the bivariate relationship between health outcomes and ecological footprint [21,25]. One reason for this difference is that there is narrow range of variation in average income levels among Canadian urban populations. Smoking rates were significantly associated with mortality-based outcomes and obesity. Variations in levels of average education figured more prominently in cardiovascular-related outcomes. Smoking is a well-known risk factor for premature mortality, reduced life expectancy, and is more common for segments of the population struggling with overweight and obesity [26,27,28,29]**.** Smoking rates and level of education are well known markers of socioeconomic status and there is preliminary evidence that groups with lower status also appropriate fewer ecological resources. Moreover, the ecological footprint is strongly correlated with income; more wealth is required to consume more resources. Results in this study may be influenced by confounding in the relationship between income and ecological footprint. The relationship between ecological footprint and life expectancy is consistent with previous research at the country level after controlling for *per capita* GDP. However, it is unclear why ecological footprint explains a significant proportion of the variation in rates of high blood pressure and is not associated with differences in health outcomes among large Canadian municipalities. 

The existence, or not, of associations between ecological footprint and health outcomes is secondary in importance to knowledge about the level of nature appropriation associated with health improvements in Canada. Globally there is a limited supply of available nature to support human needs, currently 1.6 ha of *per capita* given a current global population of just over seven billion. Supplies of nature, or the natural materials required to create wealth and support human needs, are more abundant in some regions than others. For example, it is estimated that Canada has 14.24 ha *per capita* of available nature, and the average consumption of 7.3 ha *per capita* in urban areas seems reasonable given availability. However, when examined from a global perspective, Canadian rates of nature appropriation far exceed that available 1.6 ha *per capita* and illustrate well the issue of global inequities in wealth, health and, in this context, distribution of ecological resources to support human needs. Clearly, the remarkable health status Canadians enjoy when examined using a global lens is unsustainable in terms of the levels of nature appropriation required to sustain health, and is unacceptable in terms of moving toward health equity for all. From a health measurement perspective it would seem prudent to begin a process of incorporating the ecological costs of achieving different health states so as to support and promote health using as few ecological resources as necessary. Moreover, several activities that may result in reducing municipal EF values, such as reducing energy and fossil fuel consumption through active transportation, reducing the consumption of processed foods and of goods in general, are also likely to be salutogenic. For example, reducing the combustion of fossil fuels used in transportation will result in fewer emissions of hazardous air pollutants and may promote physical activity.

The results of our analysis raise the question of whether a society would be able to improve health without appropriating nature in an unsustainable manner. Many of the healthiest societies globally are also the most unsustainable [21]. As the consumption of natural resources is also associated with income it would also be desirable to find examples health has been de-coupled from income levels. For example, Cuba, Jamaica, and Panama are three countries in the top quintile of global health-adjusted life expectancy with *per capita* ecological footprints at or below the amount required to sustain the current global population (EF = 1.8). Countries with similar values of life expectancy, but with EF values above 1.8 hectares, include Costa Rica (2.0), Uruguay (2.1), Chile (2.2), Argentina (2.2), and Mexico (2.4). The rate of natural capital consumption, as measured using EF, is associated with climate, and possibly some bioregional characteristic of Latin American lifestyles that may also contribute to longevity. This finding is consistent with theories from human ecology that incorporate biophysical factors, such as climate and biogeography, as contexts in which social factors drive environmental impacts [30].

Despite the observed relationships between EF and two population level health measures, several important aspects of the study should be noted. First, the study is exploratory as there exists no formal protocol for diagnosing a “sick” environment, or an environment of unsustainable nature appropriation. We opted to use the ecological footprint indicator as a measure of natural capital appropriation. Despite the widespread use of the EF as measure of nature appropriation, there has been some concern about its ability reflect accurately levels of appropriation at smaller geographic scales. This is not necessarily an issue with the calculation of the EF itself; rather, the data required to perform the calculation is not always available at more regional and local scales and proxy data are sometimes required to generate estimates. For example, in our study, the variability of EF values are in part a reflection of the diversity of fuel sources used to generate electricity and heat buildings. Cities that generate energy from fossil fuel sources generally have higher EFs than cities using hydroelectricity. This is an issue of concern because recent literature suggests that the production of hydroelectricity negatively impacts the environment, an effect that is not considered in the calculation of EFs [31]. The EFA measure also does not consider the local quality of natural capital and global trade. Wealthier countries and regions can afford to import foreign natural capital instead of appropriating local resources. This strategy preserves local natural capital while exploiting natural capital in poorer regions. Second, the present study is ecological in design and lacks more thorough investigation of the mechanisms linking nature appropriation to population health outcomes. The conclusions derived from the analysis hold only for city-level comparisons and do not extend to the individuals living within them. Future research is needed to elucidate the mechanisms and processes through which the appropriation of natural resources translate into changes in population health, particularly for specific health outcomes.

## 5. Conclusions

As population and development pressures increase the appropriation of nature, researchers will need to consider seriously the ecological dimensions and determinants of population health [32]. Further research in this field should explore multiple indicators for assessing natural capital appropriation, and should incorporate several different health indicators, such as psychological well-being and self-rated health. Studies should also investigate how nature appropriation and health are modified by the social determinants of health and share examples of good health achieved at more sustainable levels of nature appropriation. For example, the research literature has suggested that income inequalities in a population are related to health outcomes [33], and the effect of income should be further examined in samples containing a more representative range of income levels. Future studies should also seek empirical evidence that unsustainable natural capital appropriation is linked to poorer human health outcomes. Such findings may influence and encourage policy change toward sustainable resource use.

## References

[1] Imhoff M.L., Bounoua L., Ricketts T., Loucks C., Harriss R., Lawrence W.T. (2004). Global patterns in human consumption of net primary production. Nature.

[2] Pauly D., Chrisensen V., Guenette S., Pitcher T., Sumaila U.R., Walters C., Watson R., Zeller D. (2002). Towards sustainability in world fisheries. Nature.

[3] Pauly D., Watson R., Alder J. (2005). Global trends in world fisheries: Impacts on marine ecosystems and food security. Phil. Trans. Boil. Sci..

[4] Sinn H.W. (2008). Public policies against global warming: A supply side approach. Int. Tax Publ. Finance.

[5] Loh J.R., Green T., Ricketts J., Lamoreux M., Jenkins V., Kapos J., Randers J. (2005). The Living Planet Index: Using species population time series to track trends in biodiversity. Phil. Trans. Biol. Sci..

[6] Huynen M., Martens P., DeGroot R.S. (2004). Linkages between biodiversity loss and human health: A global indicator analysis. Int. J. Environ. Health Res..

[7] Wright S.J., Muller-Landau H.C. (2006). The future of tropical forest species. Biotropica.

[8] Wackernagel M., Rees W. (1995). Our Ecological Footprint: Reducing Human Impact on the Earth.

[9] Wiedmann T., Barrett J. (2010). A review of the ecological footprint indicator—Perceptions and methods. Sustainability.

[10] Kitzes J., Peller A., Goldfinger S., Wackernagel M. (2007). Currents methods for calculating national ecological footprint accounts. Sci. Environ. Sustain. Soc..

[11] Rice J. (2007). Ecological unequal exchange: International trade and uneven utilization of environmental space in the world system. Soc. Forces.

[12] Moran D., Wackernagel M., Kitzes J., Goldfinger S., Boutaud A. (2008). Measuring sustainable development-nation by nation. Ecol. Econ..

[13] Miller P., Rees W.E., Pimentel D., Westra L., Noss R.F. (2000). Itroduction. In Ecological Integrity: Integrating Environment, Conservation, and Health.

[14] Hu D., Huang S.L., Feng Q., Li F., Zhao J.J., Zhao Y.H., Wang B.N. (2009). Relationships between rapid urban development and the appropriation of ecosystems in Jiangyin City, Eastern China. Landscape Urban Plan..

[15] Dietz T., Rosa E.A., York R. (2007). Driving the human ecological footprint. Front. Ecol. Environ..

[16] Kitzes J., Wackernagel M., Loh J., Peller A., Goldfinger S., Cheng D., Tea K. (2008). Shrink and share: Humanity’s present and future Ecological Footprint. Phil. Trans. Boil. Sci..

[17] Ecob R., Smith G.D. (1999). Income and health: What is the nature of the relationship?. Soc. Sci. Med..

[18] Wagstaff A., van Doorslaer E. (2000). Income inequality and health: what does the literature tell us?. Ann. Rev. Public Health.

[19] Mellor J.M., Milyo J. (2001). Reexamining the evidence of an ecological association between income inequality and health. J. Health Polit. Pol. Law.

[20] Marmot M. (2002). The influence of income on health: Views of an epidemiologist. Does money really matter? Or is it a marker for something else?. Health Affair..

[21] Rainham D.G., McDowell I. (2005). The sustainability of population health. Popul. Environ..

[22] Sieswerda L.E., Soskolne C.L., Newman S.C., Schopflocher D., Smoyer K.E. (2001). Toward measuring the impact of ecological disintegrity on human health. Epidemiology.

[23] Wilson J., Anielski M. (2008). Ecological Footprint of 18 Canadian Municipalities.

[24] Jacobson S.H., King D., Yuan R. (2011). A note on the relationship between obesity and driving. Transport Pol..

[25] Wilkinson R.G., Pickett K.E. (2006). Income inequality and health: A review and explanation of the evidence. Soc. Sci. Med..

[26] Stewart S., Cutler D., Rosen A. (2009). Forecasting the effects of obesity and smoking on U.S. life expectancy. New Engl. J. Med..

[27] Centers for Disease Control and Prevention (2008). Smoking attributable mortality, years of potential life lost, and productivity losses in the United States, 2000–2004. Morb. Mortal Wkly. Rep..

[28] Jha P. (2009). Avoidable global cancer deaths and total deaths from smoking. Nat. Rev. Cancer.

[29] Chiolero A., Faeh D., Paccaud F., Cornuz J. (2008). Consequences of smoking for body weight, body fat distribution, and insulin resistance. Am. J. Clin. Nutr..

[30] Rainham D.G., McDowell I., Krewski D., Soskolne C., Westra L., Kotzé L., Mackey B., Rees W., Westra R. (2007). A Sense of Possibility: What Does Governance for Health and Ecological Sustainability Look Like?. Sustaining Life on Earth: Environmental and Human Health Through Global Governance.

[31] Environmental Defense (2001). Study Shows Hydropower is not Pollution Free. www.environmentaldefense.org/article.cfm?contentid=288.DAte.

[32] McMichael A.J., Pimentel D., Westra L., Noss R.F. (2000). Global Environmental Change in the Coming Century: How Sustainable are Recent Health Gains?. Ecological Integrity: Integrating Environment, Conservation, and Health.

[33] Judge K., Paterson I. (2001). Poverty, Income Inequality and Health. Treasury Working Paper.

